# Toward a mechanistic understanding of “peat collapse” and its potential contribution to coastal wetland loss

**DOI:** 10.1002/ecy.2720

**Published:** 2019-04-26

**Authors:** Lisa G. Chambers, Havalend E. Steinmuller, Joshua L. Breithaupt

**Affiliations:** ^1^ Aquatic Biogeochemistry Laboratory Department of Biology University of Central Florida 4000 Central Florida Boulevard BIO 302 Orlando Florida 32816 USA

**Keywords:** carbon balance, coastal peatland, coastal wetlands, mangrove, peat collapse, salt marsh, sea level rise, soil elevation loss, subsidence, wetland loss

## Abstract

Coastal wetlands are susceptible to loss in both health and extent via stressors associated with global climate change and anthropogenic disturbance. Peat collapse may represent an additional phenomenon contributing to coastal wetland loss in organic‐rich soils through rapid vertical elevation decline. However, the term “peat collapse” has been inconsistently used in the literature, leading to ambiguities regarding the mechanisms, timing, and spatial extent of its contribution to coastal wetland loss. For example, it is unclear whether peat collapse is distinct from general subsidence, or what biogeochemical changes or sequence of events may constitute peat collapse. A critical analysis of peer‐reviewed literature related to peat collapse was supplemented with fundamental principles of soil physics and biogeochemistry to develop a conceptual framework for coastal wetland peat collapse. We propose that coastal wetland peat collapse is a specific type of shallow subsidence unique to highly organic soils in which a loss of soil strength and structural integrity contributes to a decline in elevation, over the course of a few months to a few years, below the lower limit for emergent plant growth and natural recovery. We further posit that coastal wetland peat collapse is driven by severe stress or death of the vegetation, which compromises the supportive structure roots provide to low‐density organic soils and shifts the carbon balance of the ecosystem toward a net source, as mineralization is no longer offset by sequestration. Under these conditions, four mechanisms may contribute to peat collapse: (1) compression of gas‐filled pore spaces within the soil during dry‐down conditions; (2) deconsolidation of excessively waterlogged peat, followed by transport; (3) compaction of aerenchyma tissue in wetland plant roots, and possibly collapse of root channels; and (4) acceleration of soil mineralization due to the addition of labile carbon (dying roots), oxygen (decreased flooding), nutrients (eutrophication), or sulfate (saltwater intrusion). Scientists and land managers should focus efforts on monitoring vegetation health across the coastal landscape as an indicator for peat collapse vulnerability and move toward codifying the term “peat collapse” in the scientific literature. Once clarified, the contribution of peat collapse to coastal wetland loss can be evaluated.

## Introduction

Coastal wetlands are highly vulnerable to sea level rise, with estimates of 22–30% global areal loss by 2100 (Nicholls et al. 1999, IPCC 2007). Occupying the intertidal ecotones between marine and terrestrial environments, coastal wetlands experience loss from multiple directions. On the seaward edge, accelerated erosion may occur with storm activity, increases in water depth, wave height, and wave power (Schwimmer 2001, Mariotti and Fagherazzi 2010), while drowning may occur where relative sea level rise rates out‐pace soil surface elevation gains (Krauss et al. 2010, Lovelock et al. 2015a). Concomitantly, steep topography and/or human infrastructure may lead to areal habitat loss or conversion of habitat type as wetlands lack the space to transgress upslope in maintenance of an optimal elevation relative to the sea (Day et al. 2008, Hussein 2009). Peat collapse may be a third phenomenon leading to coastal wetland loss, acting vertically to convert a wetland platform to shallow open water or mudflat.

**Figure 1:**
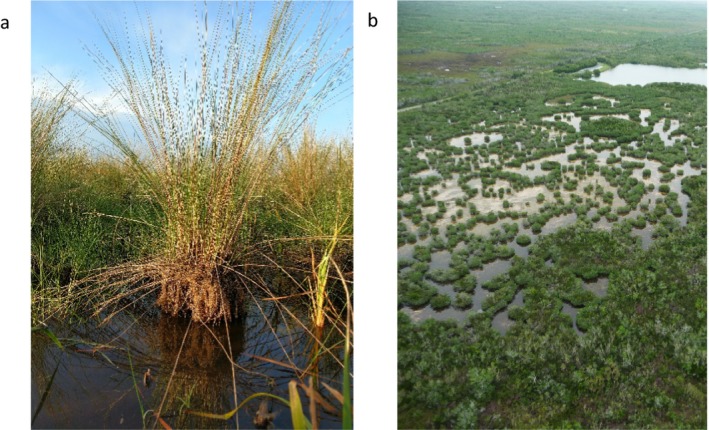
Examples of what other researchers have referred to or described as “peat collapse” in coastal wetlands: (a) a culm of *Muhlenbergia capillaris* with approximately 20 cm of exposed roots above the soil surface during the dry season, suggesting the surrounding soil collapsed (Fakahatchee Strand State Park, Florida, USA), and (b) break‐up of the wetland platform and development of interior ponds in a region exposed to excessive flooding due to hydrologic modifications (Everglades, Florida, USA). Photo credits: L. G. Chambers.

The concept of collapsing soils in both inland (Franzén 2006, Kool et al. 2006, Abbott and Jones 2015) and coastal (Delaune et al. 1994, Krauss et al. 2014, 2018, Lang'at et al. 2014, Lane et al. 2016) wetlands has appeared sporadically in the scientific literature over the past ~30 yr. For example, within the inland wetland literature, Kool et al. (2006) utilized this terminology to describe the observed loss of the “dome‐shape” in raised bogs following logging and drainage, while Abbott and Jones (2015) discussed the collapse of permafrost soils due to thawing. Within this paper, the focus is exclusively on the use of the term “peat collapse” in reference to coastal wetland loss, which is becoming increasingly common since its first use by Delaune et al. (1994). Despite its growing prevalence, the term “peat collapse” has yet to be formally defined; rather, it has been colloquially coined in the coastal wetland literature to describe the general process of interior wetland elevation loss that contributes to submergence in organic‐rich coastal marsh and mangrove ecosystems. For example, regions where exposed roots are seen above the soil platform or where interior ponds form and coalesce have been referenced by others as examples of peat collapse in coastal wetlands (Fig. 1).

With the risks of coastal wetland loss due to erosion, drowning, and a lack of space for landward transgression already substantial, it is critical to delineate how peat collapse differs from other processes of loss in coastal wetlands, such as subsidence or erosion, and consider if it may be a significant contributor to areal loss. For example, in the conterminous USA, coastal wetlands are being lost at a rate of roughly 324 km^2^ annually (Dahl 2011), with 1,704 km^2^ lost in Louisiana alone between 1978 and 2000 (Barras et al. 2003). This represents an important loss of habitat and coastal resources, while also exposing a vast reservoir of previously sequestered soil carbon (C) and nutrients to oxygenated coastal waters. Aerobic aquatic environments, such as shallow bays and ponds, can increase the potential for soil C mineralization (conversion to CO_2_) and nitrogen (N) and phosphorus (P) release (Steinmuller et al. 2018). Implications include a climate feedback that has not yet been accounted for in global and regional C models, additional dissolved inorganic C (DIC) within the coastal zone that could contribute to pH shifts, and increased potential for coastal eutrophication with the additional source of N and P.

The purpose of this article is to (1) discuss the meaning of the term “peat collapse,” (2) examine the peer‐reviewed literature related to peat collapse, (3) critically evaluate the possible mechanisms leading to peat collapse in coastal wetlands, (4) develop a conceptual framework of the most likely scenarios for wetland loss via peat collapse, (5) present a working definition of coastal wetland peat collapse, and (6) suggest future research directions and management implications. Scientists and resource managers can only evaluate the ecological ramifications and determine the best management practices for remediating collapsed/collapsing wetlands after a consensus has been reached on what constitutes peat collapse, how it occurs, and how to correctly identify it in the field.

## Semantics of “Peat Collapse”

When searching for peer‐reviewed literature relevant to coastal wetland peat collapse, the inconsistency in the use of the term is readily apparent. Only five publications were found using the term “peat collapse” in reference to their original research findings (Delaune et al. 1994, Cahoon et al. 2003, 2004, Wigand et al. 2014, Krauss et al. 2018), although several others referenced the term somewhere within the manuscript (e.g., Portnoy and Giblin 1997, Smith et al. 2009, DeLaune and White 2012, Chambers et al. 2014). The most commonly encountered alternative term was “subsidence,” defined as soil elevation loss. So, what is peat collapse, and is it unique from subsidence? To answer this, we must first evaluate the meaning of the terminology.

Beginning with the explicit definition of the terms, the first word, “peat,” refers to organic soils (>20–30% organic matter by mass) dominated by undecomposed to slightly decomposed plant tissue (Brady and Weil 2004, Reddy and DeLaune 2008). Within the peat collapse literature, the term “peat” has been used to indicate an organic‐rich soil in a loose context, as reported organic matter contents range from 18% to 65%, and the extent of decomposition is never mentioned (e.g., Portnoy and Giblin 1997, Day et al. 2011). The second part of the term, the verb “collapse,” means to “fall or shrink together abruptly and completely,” “to cave or fall in or give way,” or “to suddenly lose force” (Merriam‐Webster, 2018). Considered in the context of wetland soils, the process of peat collapse suggests three key concepts: (1) a loss of elevation in organic‐rich soils (falling together or caving in), (2) this elevation loss is related to a reduction in soil strength or structural integrity (losing force), and (3) the phenomenon occurs suddenly or abruptly. Early work referencing peat collapse made a clear distinction between erosive processes leading to wetland loss, and a rapid elevation decline associated with in‐situ biogeochemical changes (Nyman et al. 1994). Therefore, throughout this review we maintain the general conceptual tenant that peat collapse is unique from coastal erosion, although erosion may play a role as a secondary driver of elevation loss.

### Soil elevation loss

Nine published studies were found quantifying elevation loss attributed to peat collapse in coastal wetlands (Table 1). Only four of these studies applied the term peat collapse to their original findings; the remaining five studies we interpreted as relevant to coastal wetland peat collapse based on our understanding of the terminology. According to this data set, total elevation loss observed during the various study periods ranged from 1.48 to 15 cm (Table 1). If values are converted into rates of a common unit (cm/yr) for comparative purposes, annual elevation losses range from approximately 1 cm/yr, up to 7.5 cm/yr, but it is acknowledged that elevation loss may be non‐linear with time (Portnoy and Giblin 1997). Surface elevation tables (SET), in conjunction with marker horizons (MH), were the most commonly cited method for evaluating elevation change; others also used benchmarks and general surveying techniques (Table 1).

**Table 1:** Key case studies found within the peer‐reviewed literature that quantify sudden soil elevation loss in organic‐rich coastal wetland soils, including both those that used the term “peat collapse” and those we interpreted as relevant to coastal wetland peat collapse without invoking the terminology

Site	Salinity regime	Collapse rate^a^ (cm/yr)	Length of study (yr)	Total elevation loss (cm)	Elevation quantification method	Source	Term used	Stressor	Driver
*Spartina* marsh, Louisiana (USA)	saline	7.5	2	15	transit level and benchmark	Delaune et al. (1994)	peat collapse	experimental manipulation (herbicide) and excessive flooding	vegetation death
Marsh (various), Louisiana (USA)	freshwater	2.8	1.5	4.2	standard surveying equipment	Lane et al. (2016)	decreased elevation	experimental manipulation (herbicide)	vegetation death
Marsh (various), Louisiana (USA)	brackish	1.0	1.5	1.6	standard surveying equipment	Lane et al. (2016)	decreased elevation	experimental manipulation (herbicide)	vegetation death
Marsh (various), Louisiana (USA)	saline	1.0	1.5	1.5	standard surveying equipment	Lane et al. (2016)	decreased elevation	experimental manipulation (herbicide)	vegetation death
Mangrove forest, Kenya	saline	3.2	2.1	5.1	RSET‐MH	Lang'at et al. (2014)	subsidence	experimental manipulation (girdling and cutting)	vegetation death
Mangrove swamp, Florida (USA)	saline	3.5	1.75	6.09	RSET‐MH	Whelan (2005)	elevation loss	lightning	vegetation death
*Spartina* marsh, Louisiana (USA)	saline	2	3	6	SET‐MH	Day et al. (2011)	collapse	excessive flooding	vegetation death
*Spartina* marsh, Louisiana (USA)	saline	0.67	3	2	SET‐MH	Day et al. (2011)	collapse	excessive flooding	vegetation death
Mangrove swamp, Bay Islands, Honduras	saline	1.1	1.3	1.4	RSET‐MH	Cahoon et al. (2003)	peat collapse	hurricane	vegetation death
Diked marshes, Massachusetts (USA)	experimental saltwater additions	7^b^	1.75	6–8	not mentioned	Portnoy and Giblin (1997)	subsidence	experimental manipulation (saltwater addition)	ND
Mangrove swamp, Florida (USA)	saline	N/A	single sampling	6–8	inferred from bulk density profiles	Krauss et al. (2018)	peat collapse	anthropogenic hydrologic alteration	vegetation stress/death
*Spartina* marsh, Texas (USA)	saline	2.8^c^	3.3	2.8	SET‐MH	Cahoon et al. (2004)	peat collapse	excessive flooding (hurricane)	vegetation death

Notes

We recognize there are other studies that directly referenced the phrase “peat collapse,” but because they did not provide quantitative evidence of decreased elevation, they could not be critically evaluated as case studies for coastal wetland peat collapse. ND, No Data; N/A, Not Applicable.

a All measurements were converted to cm/yr to create a common unit by which to compare rates. However, it should be noted that soil elevation loss is likely a non‐linear process with time and any rates presented do not lend themselves to the assumption that rate or direction of change will continue in the future.

b Authors report elevation loss occurred in first 6 months, with no subsequent loss as time progressed to 21 months.

c Authors report elevation loss occurred in first 5 months, then slightly recovered in the subsequent 7 months.

### Reduced soil strength

Quantification of a loss in soil strength or structure is also limited in the peat collapse literature and often does not co‐occur with measurements of soil elevation, hindering our ability to connect these two concepts as key foundations of peat collapse. Approaches for assessing changes in soil structure and strength focus on (1) identifying changes in soil bulk density (i.e., mass per volume) and (2) the use of geotechnical engineering tools, such as shear stress vanes (Whelan 2005, Schultz et al. 2016) and cone penetrometers (Day et al. 2011, Twohig and Stolt 2011) to assess changes in load‐bearing capacity and compressibility of soils. If soil structure is compromised, allowing the soil to consolidate, then soil bulk density should increase at the bottom of the collapse zone as previously existing pore space is replaced by the overlying solid material. This increase in surface or shallow subsurface bulk density is commonly found in collapsed inland peat domes (Franzén 2006, Kool et al. 2006) and in slumping permafrost (Abbott and Jones 2015), but results from coastal wetlands are inconsistent. Portnoy and Giblin (1997) observed increased bulk density in some depth segments (e.g., 20–40 cm) of soil cores demonstrating elevation loss, as did Lang'at et al. (2014). However, others have found no relationship between elevation loss and bulk density, instead suggesting the soil material may have been washed away following the collapse, or rapidly oxidized (Delaune et al. 1994, Day et al. 2011). We propose that this inconsistency may be a result of hydrology, such that sites undergoing regular dry‐down (e.g., due to seasonality, drought, or a large tidal range) are likely to experience pore space compression leading to greater bulk densities. Conversely, sites that remain waterlogged are likely to experience excessive swelling or dilation of the soil organic matter (SOM; see *Pore space compression*), potentially resulting in elevation loss due to the sloughing and transport of this loose, unconsolidated material.

Significantly lower soil shear strength in deteriorating wetlands relative to reference wetlands has been observed, particularly in the root zone (Day et al. 2011, Twohig and Stolt 2011, Wigand et al. 2014). Further study indicates that measures of soil shear strength are strongly correlated with live belowground biomass (Sasser et al. 2018). Because roots form the scaffolding that holds highly porous organic soils together, dead, dying, or stressed vegetation that is not allocating biomass belowground can weaken the soil structure within the root zone.

### Abrupt event

The definition of “collapse” indicates an abrupt, rapid, or sudden process. Unfortunately, these descriptors are subjective based on the scientific field of study: for biogeochemists and ecologists, abrupt or rapid events occur over sub‐diurnal or seasonal scales, while for geologists, changes in coastal geomorphology are considered abrupt if they occur over centennial or millennial timescales (Cowell and Thom 1994). Of the existing studies providing a quantification of soil elevation loss rate, all studies spanned approximately three years or less (Table 1). This is concerning considering elevation is known to exhibit temporal fluctuations and even periodicity based on seasonal changes in hydrology, storm events, and other factors (Cahoon 2006, Whelan et al. 2009, Cahoon et al. 2011, Breithaupt et al. 2018). In order to classify a loss of surface elevation as something unusual, possibly even as a collapse event, it is necessary to have a record of previous elevation trends and fluctuations for comparison. The beginning of the record cannot be assumed to be temporally neutral; it may instead represent a high, middle or low account of surface elevation over time. Therefore, a period of declining or neutral vertical development may simply be a return to equilibrium rather than a collapse or drowning event.

Furthermore, several studies measured elevation only at the start and end of the study (with no observations over the intervening months or years), making it difficult to identify the precise timescale when the elevation loss occurred. The most rapid rates of elevation loss published are ~2.8 cm within 5 months in an excessively flooded Texas marsh (Cahoon et al. 2004) and 6–8 cm within 6 months in intact freshwater marsh cores treated with seawater (Portnoy and Giblin 1997), while most other rates are on the order of approximately 1–3 cm/yr (Table 1). For context on how these rates compare to related ecological phenomena, even the lowest published annual rate of elevation loss (1.0 cm/yr) is still more than three times greater than the rate of eustatic sea level rise (Church et al. 2013), but within the range (0–2.2 cm/yr) of globally documented coastal wetland accretion rates (Breithaupt et al. 2018). Therefore, whether these annual rates of elevation decline are significant enough to create a deficit in elevation capital that shifts the ecosystem toward conversion to open water depends on local rates of sea level rise, the persistence of the elevation loss, and the response of vegetation (Reed 2002, Cahoon and Guntenspergen 2010, Lovelock et al. 2015b, Cahoon et al. 2019).

## Evidence of Peat Collapse in Coastal Wetlands

For the purposes of this review, some subjectivity was involved in the identification of studies that we interpreted as relevant to peat collapse, even if the term was not presented in the text. In particular, we sought studies located in highly organic coastal wetland soils that documented (quantitatively) some type of non‐erosional (often interior wetland) soil elevation loss. The majority of studies we evaluated were identified by starting with a published paper that made clear reference to “peat collapse” (Delaune et al. 1994, Cahoon et al. 2003, Day et al. 2011) and searching multiple iterations of papers citing, and cited by, these keystone papers.

### Experimental evidence

Delaune et al. (1994) were first to use the term “peat collapse” in reference to coastal wetland submergence via pond formation. During a 2‐yr study period, 20 hummocks containing live *Spartina alterniflora* died due to manipulation (herbicide treatment) and excessive flooding. Measurements before and after the treatment revealed a 9–15 cm decrease in hummock surface elevation. Additionally, a reduction in soil depth above the ^137^Cs 1963 depth horizon without a corresponding loss of the ^137^Cs inventory indicated that the volume loss was non‐erosional. Rather, the authors suggest the elevation loss was due to the “collapse of the living root network,” possibly in combination with accelerated decomposition and loss of root turgor and porosity (Delaune et al. 1994). Follow‐up field experiments by Lane et al. (2016) treated plots of fresh, brackish, and saline marshes with herbicide and documented mean elevation loss in treated plots of 4.24, 1.56, and 1.48 cm, respectively, within 1.5 yr. This study found no accompanying change in litter decomposition rates in the treated plots.

In a Kenyan mangrove forest, girdled and then cut *Rhizophora mucronata* plots showed a mean elevation loss of 5.1 cm, while untreated plots gained 1.1 cm in elevation (Lang'at et al. 2014). In the first ~5 months of this study, both CO_2_ and CH_4_ flux were higher from girdled plots relative to control plots. At the conclusion of the 2‐yr study, treated plots also showed higher decomposition rates, soil temperatures, and bulk density. The elevation loss in this study was attributed to “collapse and decomposition of dying roots and sediment compaction” (Lang'at et al. 2014). Finally, Portnoy and Giblin (1997) documented 6–8 cm of “sediment subsidence” in the first 6 months following plant death in an intact soil core study with freshwater wetland soils exposed to artificial seawater.

### Observational evidence

Non‐manipulative studies of peat collapse generally focus on the impacts of acute disturbances, or investigations of coastal wetland loss over time, often referencing a chronic disturbance such as sea level rise or hydrologic manipulation. Moreover, most observational studies do not directly quantify elevation change, but simply note wetland loss, making it difficult to assess if peat collapse was involved in the submergence of the wetland platform.

Hurricanes and accompanying vegetation death produced an elevation loss of 1.1 cm/yr during a 15 month study in Honduras (Cahoon et al. 2003). Study plots experiencing the highest impact (complete mangrove mortality) showed zero root production and significantly lower soil shear strength between 0 and 15 cm, relative to low impact plots (Cahoon et al. 2003). Hurricanes may also work synergistically with other stressors (saltwater intrusion, erosion) to cause interior coastal wetland loss. For example, in the southwestern Everglades (Florida, USA), a series of historic “collapse” events are implicated in converting interior marsh to present‐day Whitewater Bay, resulting in the loss of 2–4 m deep peat deposits across ~140 km^2^ (Wanless and Vlaswinkel 2005). This loss was attributed to a combination of storm damage and hydrologic modification (Wanless and Vlaswinkel 2005). Moreover, elevation loss has also been noted in mangrove canopy gaps created by lightning strikes (Sherman et al. 2000, Whelan 2005).

Sea level rise, and accompanying changes in salinity and inundation, is a commonly cited cause of peat collapse, representing a chronic stressor. For example, extensive interior wetland submergence (ponding) has been observed in coastal Louisiana (USA), where several studies document the rapid loss of large areas of marsh, despite an adequate sediment supply and negligible wave energy (Barras et al. 2003, Day et al. 2011). One 3‐yr study documented the break‐up and submergence of a marsh exposed to tidal inundation 85% of the time, while a nearby reference marsh that was only inundated 15% of the time remained stable (Day et al. 2011). Similar scenarios have been observed in Jamaica Bay marshes (New York, USA) where excessive waterlogging is coincident with deterioration of the marsh platform, creating unvegetated ponds of unconsolidated “soupy” soils (Hartig et al. 2002, Wigand et al. 2014, Cahoon et al. 2019). More recently, long‐term hydrologic changes associated with road construction have been implicated in mangrove death and suspected elevation loss (Krauss et al. 2018).

## Mechanisms of Coastal Wetland Peat Collapse

To date, studies investigating peat collapse in coastal wetlands have focused almost exclusively on *drivers*, what we define herein as the causes, or “why” peat collapse is occurring. Of the nine key coastal peat collapse papers identified, vegetation stress and/or death was the only driver explicitly mentioned as causing the elevation loss, with a variety of different stressors producing the decline in vegetation health (Table 1). Although this relationship between the biological driver (plant death) and the physical change (elevation loss) presented in the literature is compelling, it is difficult to identify the true cause‐and‐effect relationship, and to determine if peat collapse is a unique process, until there is an understanding of the *mechanisms* of peat collapse. We define mechanisms as “how” peat collapse occurs. More specifically, to produce a loss in soil elevation there are only two possible physical explanations‐ the mass of the soil remains the same, but the volume decreases (i.e., compaction or compression of some kind occurs), or the mass of the soil itself decreases (i.e., previously existing material is removed, either through transport or transformation).

### Properties of coastal wetland peat

Coastal wetland peat, like all soils, is composed of three components: mineral (inorganic) material, organic matter, and pore spaces (filled with gas and/or water). The contribution of mineral material to coastal wetland soils tends to increase with proximity to the coast, tidal creeks, and/or rivers, while wetland areas that are further removed from high‐energy hydrologic exchanges are typically dominated by organic‐rich soils originating from undecomposed plant litter (Odum 1988). These lower‐energy, often times interior wetland areas, are the regions of coastal wetlands where soils are most organic and peat collapse has typically been documented or implicated (e.g., “interior ponding”; Penland et al. 2000, Hartig et al. 2002). An exceptionally high percentage of pore space per volume in peat soils contributes to a low bulk density and high hydraulic capacity. Often, 75–95% of the volume of peat soils is composed of voids, with the solid matrix being held together by nothing more than fibrous detritus and interwoven mats of live roots (Kaye and Barghoorn 1964, Nyman et al. 1990, Day et al. 2011). Based on these physical properties, peat collapse can only occur if one or more of the physical elements of the soil (pore space, organic matter, or mineral matter) experiences a reduction in volume, mass, or both.

### Pore space compression

The exceptionally high contribution of pore spaces to the overall volume of organic coastal wetland soils may suggest a reduction in the void space occupied by gas and/or water is an obvious mechanism for peat collapse. However, upon further evaluation, there is one critical caveat to the compression of pore space as a viable and widespread explanation: under normal temperature and pressure, water is essentially incompressible (Fine and Millero 1973). In wetland soils, especially highly organic ones, the vast majority of pore space is often permanently occupied by water. Studies have shown the water holding capacity of soil organic matter is so high, anaerobic conditions can remain even during periods of dry‐down or low tide (Chambers et al. 2013). It is important to note that, under flooded conditions, a small aerated layer can persist near the soil surface and undergo degassing and compression under the weight of a significant overburden, but this elevation loss is typically temporary, followed by re‐gasification (Chapman 1974, Cahoon 2006). In order for any significant, permanent pore space compression to occur, the soil must undergo a period of drying sufficient to increase the proportion of gas‐filled pore space. Once filled with gases, gravity‐driven compaction of the solid components in peat soils is possible, particularly in the absence of live roots that normally provide structural support (Kaye and Barghoorn 1964). The role of drought or draining in initiating peat collapse has not been mentioned in previous coastal wetland studies, but is regularly implicated as a mechanism for the collapse of inland raised bogs (Franzén 2006, Kool et al. 2006, Couwenberg et al. 2010).

More commonly in coastal wetlands, excessive flooding is cited in the literature as a stressor associated with peat collapse (Table 1). Excessive, continuous flooding can dramatically alter organic soil structure, usually through the dilation and swelling of the organic material (Nuttle et al. 1990, Whelan et al. 2005, Cahoon et al. 2011). A lack of periodic desiccation also prevents the incorporation of new sediment and detritus into the soil structure, which occurs through a reduction in pore space volume upon dry‐down (Day et al. 2011). Under conditions in which excessive flooding is coupled with vegetation death (i.e., the loss of live roots to hold waterlogged material together), peat soils would be destabilized, potentially dispersing to become an unconsolidated flocculent layer above the soil surface. Under these conditions, the loose unconsolidated layer could be easily removed with low energy waves or currents (Day et al. 2011). These processes were observed in mangrove peat soils experimentally exposed to increased inundation, resulting in a decrease in surface soil bulk density as water‐logged peat sloughed‐off during ebb tides (Chambers et al. 2014).

### Soil organic matter compaction and transformation

Soil organic matter comprises everything below the soil surface of biotic origin: fresh, degraded, and humified plant and animal matter, live and dead roots, and soil micro‐ and macro‐organisms (Reddy and DeLaune 2008). Root biomass (both live and dead) can represent up to 22% of the total mass of soil solids in coastal peatlands (Sasser et al. 2018) and typically holds together the largest pool of SOM–plant‐litter, in various phases of decay. To contribute to a quantifiable elevation loss, SOM must compact (decrease in vertical volume) or transform (e.g., change state, such as through mineralization). Compaction could occur as a physical response to an increase in overburden on the soil surface, such as the deposition of wrack material or a storm surge (Cahoon 2006, Whelan et al. 2009), or simply the compaction of peat under its own weight. The latter is referred to as “autocompaction” and describes the slow settling and compression of peat increasing with depth (Kaye and Barghoorn 1964), rather than a surficial, abrupt process like peat collapse.

A more probable mechanism for abrupt elevation change in coastal wetlands is the compaction of void spaces within the living root structures of wetland vegetation, concurrent with plant death or severe stress. Nearly all wetland plants possess aerenchyma, internal gas‐filled spaces that assist in gas storage and movement through the roots and shoots (Cronk and Fennessey 2001). Plant stressors, such as increased salinity, can affect the osmotic potential of the roots, causing them to decrease turgor (Volkmar et al. 1998), while plant death results in a complete loss of turgor and collapse of the gas‐filled aerenchyma. These gas spaces within the roots can comprise a significant volume of the soil, up to 57% of the cross‐sectional area of mangrove roots (Pi et al. 2009). The connection between aerenchyma collapse and elevation loss has not been directly tested, but has been previously referenced as a plausible mechanism for peat collapse (Delaune et al. 1994, Lang'at et al. 2014) and would be expected to produce a sudden change in soil volume.

Mineralization is the final step in the decay process and results in the conversion of organic compounds to inorganic molecules during microbial respiration, with CO_2_ being a dominant end product (Melillo et al. 1989). As such, mineralization removes mass from the SOM pool through conversion to a gas. Under healthy wetland conditions, mineralization losses are more than offset by a constant input of new C, mainly through photosynthesis, which is so efficient at sequestering C that wetland ecosystems typically function as net C sinks (Chmura et al. 2003, Reddy and DeLaune 2008). However, if primary productivity declines or vegetation death occurs as a result of an acute or chronic stressor, mineralization would continue, with the potential to shift the C balance of the system toward a net C source (Wilson et al. 2018). This concept has been demonstrated by studies of net ecosystem exchange (NEE) following a major disturbance, such as a fire, in which a system that normally functions as a net C sink rapidly shifts to a net C source when photosynthetic C inputs cease (Beringer et al. 2003). Moreover, using mean nighttime respiration rates from an unvegetated south Florida mangrove peat soil core as a proxy for NEE following complete vegetation death suggests all of the C in the upper 2 cm of soil (representing 20% of the mass of total solids) could be mineralized in approximately 67 d in the absence of C inputs (assuming a constant decay rate; data from Chambers et al. 2014). A mass loss of this magnitude is likely to undermine soil structure and could contribute to an abrupt elevation loss, demonstrating that live vegetation not only preserves soil integrity with the supportive root network, but also by offsetting soil mineralization losses.

In organic wetland soils, mineralization rates are generally limited by the rate of oxygen (O_2_) penetration into waterlogged soils, resulting in the reliance on slower, less efficient microbial respiration pathways (e.g., nitrate [NO_3_^−^] reduction, sulfate [SO_4_^2−^] reduction, methanogenesis; Reddy and DeLaune 2008). In some circumstances, a lack of simple, easily degradable organic compounds can also slow mineralization (Alvarez and Alvarez 2000). To accelerate mineralization to the extent that a sudden and significant decline in SOM mass occurs and produces a decrease in elevation, a major shift must occur in (1) the abundance of labile, easily degradable organic compounds (i.e., electron donors) or (2) the availability of one or more electron acceptors (e.g., O_2_, NO_3_^−^, SO_4_^2−^).

Plant roots are known to contain and exude an abundance of simple C compounds (e.g., amino acids, sugars, organic acids, secondary metabolites; Bais et al. 2006) that could stimulate SOM mineralization rates upon death. This would likely be seen as a sudden, rapid increase in CO_2_ production as soil microbial community size and turnover rate increases in response to the new energy source, followed by a linear increase in CO_2_ production over weeks to months (Kuzyakov 2010). Similarly, an increase in the availability of electron acceptors may also stimulate mineralization rates. Here, we will focus on O_2_, NO_3_^−^, and SO_4_^2−^ additions, as these are often the most abundant and well‐studied electron acceptors in coastal wetlands.

Dry‐down or drought conditions in wetlands allow for increased penetration of O_2_ into the soil, significantly accelerating CO_2_ production. Research on non‐coastal peat dome collapse suggests anywhere from 35% to 100% of elevation loss following artificial drainage is caused by oxidation (Couwenberg et al. 2010). In coastal wetlands, significant de‐watering is less likely, but soils may still be exposed to O_2_ dissolved in the water column, such as an influx of oxygenated seawater along the wetland edge, or a newly formed open water pond. For example, a laboratory study mimicking the introduction of aerobic coastal waters into a brackish marsh soil found a 66% increase in CO_2_ production (Steinmuller et al. 2018). Extensive investigations have addressed the impacts of NO_3_^−^ loading (or NO_3_^−^ + phosphate) on coastal wetland systems, which combines the addition of an alternative electron acceptor with commonly limiting nutrients. Outcomes of these fertilization experiments are often site specific. Several have found accelerated decomposition rates and/or CO_2_ flux rates under fertilization (Morris and Bradley 1999, Wigand et al. 2009, Deegan et al. 2012), while others indicate no change in decomposition rate or soil strength (Graham and Mendelssohn 2014). It is also noteworthy that nutrient addition can reduce belowground biomass allocation, which can contribute to reduced soil strength (Turner 2011, Castañeda‐Moya et al. 2013).

The electron acceptor that has received the most attention as being potentially linked to coastal wetland peat collapse is an increase in the abundance of SO_4_^2−^, which is typically associated with saltwater intrusion and sea level rise. Several studies confirm a short‐term acceleration in CO_2_ flux in freshwater and oligohaline wetland soils exposed to seawater (containing SO_4_^2−^; Chambers et al. 2011, 2013, Wang et al. 2017), but the stimulation of SOM mineralization does not appear to persist (Neubauer et al. 2013). In field manipulations where freshwater tidal marshes were dosed with seawater (~2–5 ppt), there was no change in CO_2_ flux within the +salt treatments in year 1 (Neubauer 2011) and the +salt treatment was significantly lower than the control or +fresh treatment by 3.5 yr (Neubauer et al. 2013). Furthermore, in coastal wetlands that are already brackish in nature, increased salinity does not appear to alter CO_2_ production rates, presumably because SO_4_^2−^ is already non‐limiting (Chambers et al. 2014).

To test if accelerated SOM mineralization due to labile C or electron acceptor addition is a plausible mechanism of peat collapse, CO_2_ flux measurements need to be paired with soil elevation monitoring. Lane et al. (2016) found that elevation loss in herbicide‐treated coastal wetlands was not accompanied by an increase in cumulative CO_2_ flux. Rather, CO_2_ flux from the freshwater treatment plot did not differ from the control plot, and the brackish and saline treatment plots had lower CO_2_ flux then their respective control plots. In contrast, Lang'at et al. (2014) observed periodically greater CO_2_ flux from impacted mangrove plots, relative to control plots. Taken together, there is limited evidence in the literature to suggest the addition of labile C or an additional electron acceptor alone (particularly NO_3_^−^ and SO_4_^2−^) would accelerate respiration significantly enough, or quickly enough, to account for abrupt measureable elevation loss in coastal wetlands. However, these additions may contribute to soil mass loss and destabilization in combination with vegetation death and other mechanisms of elevation loss.

### Mineral matter compaction and dissolution

Based on the nature of peat soils, inorganic mineral matter should comprise no more than 4–20% of the soil volume (Kaye and Barghoorn 1964, Nyman et al. 1990, Brady and Weil 2004). Changes in mineral matter that may contribute to a decline in surface elevation include compaction and dissolution, since erosive transport is not compatible with the working definition of peat collapse (Nyman et al. 1994). Coarse textured materials (e.g., sand, gravel, and larger) resist compression once the grains have settled into a tightly packed arrangement, whereas crystalline clays can experience significant compression due to their high porosity and layered structure (Brady and Weil 2004). The compaction of fine‐grained sediments is considered a major contributor to coastal wetland submergence, particularly in deltas, but is typically documented as a deep, gradual process of subsidence (Coleman et al. 1998, Meckel et al. 2007). Dissolution of mineral matter may be significant in carbonate coastal ecosystems (e.g., Florida Everglades [USA], Yucatan Peninsula, Australia, and the Arabian Peninsula) where carbonates contribute substantially to coastal wetland soil volume (Breithaupt et al. 2017, Saderne et al. 2018). Carbonic acid formed during microbial respiration and root excretions, and nitric or sulfuric acids formed during nitrogen and sulfur cycling could drive the pH low enough to dissolve carbonate substrates, as observed in seagrass beds of Biscayne Bay, FL (Zieman 1972). However, because of the small soil volume represented by mineral matter in peat soils, we do not anticipate changes in this portion of the soil to be a significant mechanism for peat collapse.

## Conceptual Framework for Coastal Wetland Peat Collapse

**Figure 2:**
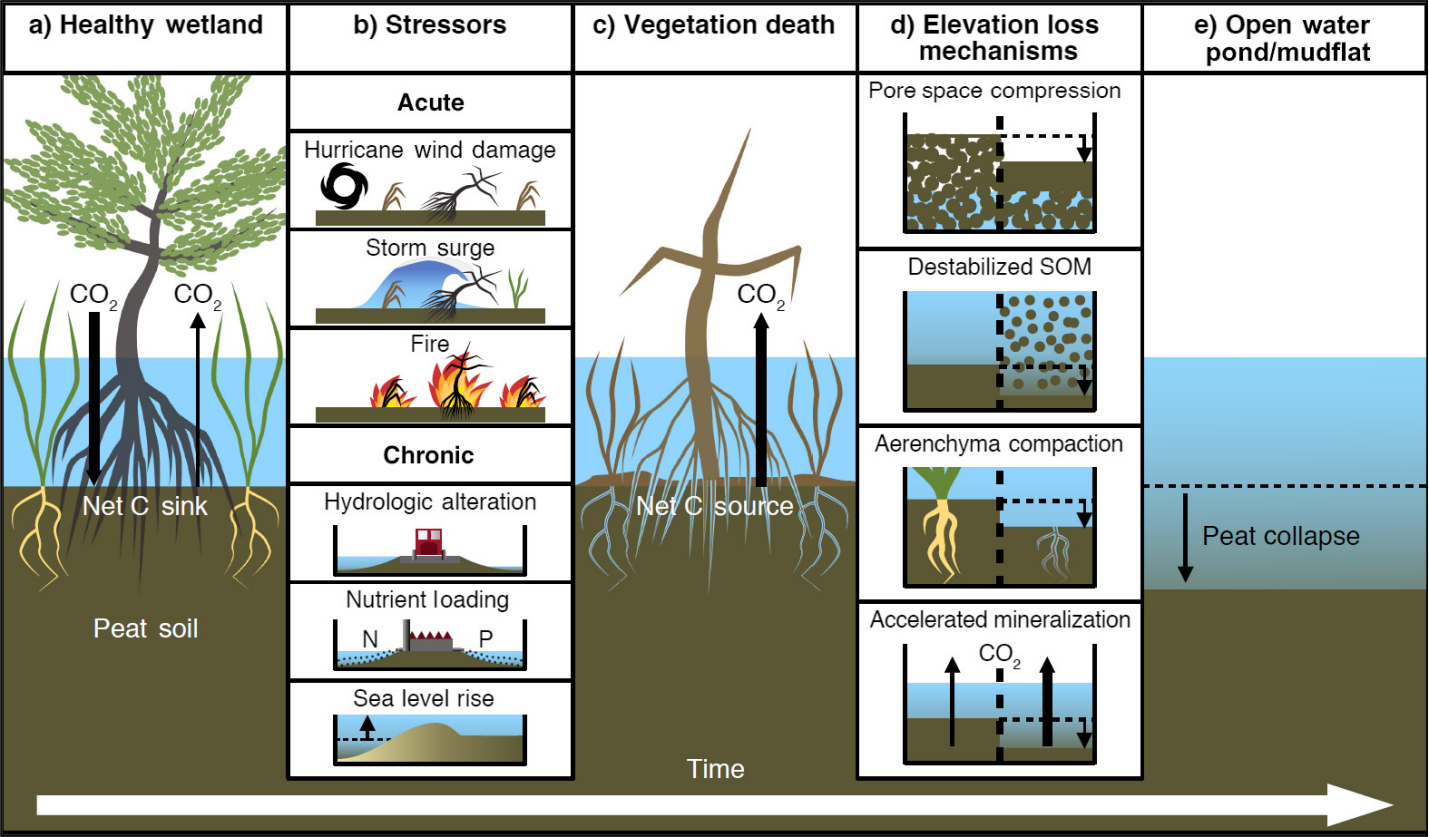
Conceptual framework detailing the potential pathways that a healthy wetland (panel a) that is exposed to various acute or chronic environmental stressors (panel b) can result in vegetation death (panel c), leading to four potential (non‐exclusive) mechanisms of soil surface elevation loss (panel d) and ultimately conversion to an open water pond or mudflat (panel e).

Based on existing literature and careful consideration regarding biogeochemical processes and the physical properties of peat soil, we present the following conceptual framework detailing the process and mechanisms of coastal wetland peat collapse (Fig. 2). Over time, healthy coastal wetlands are exposed to a variety of acute and chronic stressors (Fig. 2a, b). If these stressors are sufficient to result in severe vegetation decline or death, the system is expected to shift from a net C sink, to a net C source, due to the loss of C sequestration by primary producers (Fig. 2c). We view vegetation death as a key tipping point in the process of peat collapse, in which the trajectory of the system may recover through natural recruitment and re‐colonization or restoration intervention, or proceed down a path of substantial soil C loss and exposure to other mechanisms of elevation decline (Fig. 2d, Table 2). Specifically, the four most probable mechanisms of elevation loss include:

**Table 2:** Characteristic properties of the four proposed scenarios for coastal wetland elevation loss via peat collapse

Scenarios for elevation loss	Vegetation death/decline	Hydrologic regime		Mechanism for elevation loss		Diagnostic soil properties	
		Periodic dry‐down	Excessive flooding	Decreased soil volume	Decreased soil mass	Increased soil bulk density	Decreased soil shear strength
1) Pore space compression	✓	✓		✓		✓	
2) Destabilized soil organic matter	✓		✓		✓		✓
3) Compaction of aerenchyma and/or root channels	✓	✓	✓	✓	✓	✓	✓
4) Acceleration of soil mineralization	✓	✓	✓		✓		✓

1. Compression of gas‐filled pore spaces during dry‐down conditions due to the loss of structurally supportive live roots to maintain soil void spaces. This scenario is most likely in systems exposed to a large tidal range, variable hydrology (e.g., definitive wet and dry seasonality), or drought conditions, and would result in decreased soil volume and increased surface bulk density.
2. Destabilization of excessively water‐logged SOM following the loss of structurally supportive live roots. This scenario is likely in systems experiencing sea level rise or increased flooding due to hydrologic modifications, and in highly organic soils composed of partially or undecomposed detritus prone to dilation and dispersion. Under these conditions, surface soils and new detrital inputs produce an unconsolidated floc characterized by low soil shear strength. This material can disperse throughout the water column, resulting in a loss of soil mass, and be susceptible to transport off‐site with minimal currents or wave energy.
3. Compaction of gas‐filled aerenchyma tissue in wetland plant roots, and possibly root channels, due to severe vegetation stress (e.g., osmotic stress due to saltwater intrusion) and/or vegetation death. This may occur under any hydrologic regime if aerenchyma and root channels retain their status as gas‐filled spaces. Elevation loss could result from a decrease in soil volume (and subsequent increase in surface soil bulk density) if the gas‐filled spaces collapse and the soil consolidates, or from a loss of soil mass (and subsequent decrease in soil shear strength) if root contraction destabilizes soil material.
4. Acceleration of soil mineralization due to the addition of a limiting electron donor (e.g., labile C from dying roots), or electron acceptor (e.g., O_2_ from drying or mixing with open water; NO_3_^−^ from anthropogenic inputs or internal cycling; or SO_4_^2−^ from saltwater intrusion). This scenario is possible under any hydrologic regime and would result in a loss of soil mass to the atmosphere, likely accompanied by decreased soil shear strength.

Once elevation loss has occurred, recovery to a healthy, vegetated wetland becomes improbable without significant management intervention. This is because the optimal position for macrophyte growth within the intertidal zone has been lost (Morris et al. 2002) and soil physical structure has been altered under all scenarios (e.g., either becoming too unconsolidated, or possibly too dense for root growth [Bengough et al. 2006]). Therefore, the loss of elevation (Fig. 2d) represents a regime shift toward an open water, “soupy” mud flat condition (Fig. 2e). We emphasize that none of these proposed mechanisms are likely to occur in isolation, and most instances of peat collapse will likely result from a combination of mechanisms and possibly even multiple stressors.

## Working Definition of Peat Collapse

Through the process of conceptualizing the mechanisms of peat collapse, we are better able to begin development of a working definition specific to coastal wetland ecosystems. We emphasize this is a “working” definition because currently available empirical data are insufficient to inform many aspects of the phenomenon; rather, we hope to begin a conversation and spur new lines of inquiry that will ultimately coalesce into a formal definition.

Based on our current understanding, coastal wetland peat collapse is a specific type of shallow subsidence unique to highly organic soils in which a loss of soil strength and structural integrity contributes to a decline in elevation, over the course of a few months to a few years, below the lower limit for emergent plant growth and natural recovery. Under this definition, the elevation loss occurs within the active root zone, or directly below it (e.g., the top ~15–50 cm, depending on plant community [Cahoon et al. 2003, Whelan 2005]). Plant stress and/or death is a necessary precursor and key trigger for the destabilization of the soil platform under this conceptualization, leaving the low‐density soil vulnerable to the additional mechanisms for elevation loss detailed in Fig. 2. We suggest the prognostic role of vegetation stress and/or death in coastal wetland peat collapse not only on the basis of the consistent reference to it as the proximate driver in published case studies (Table 1), but also the logic that the existence of healthy vegetation coverage means the maintenance of a strong root network to support the soil matrix and maintain its’ strength, effectively preventing the initiation of a loss in strength implied by the term “collapse.” Due to the heterogeneity of vegetation and micro‐habitats within coastal wetlands, we expect peat collapse will initially manifest as a “patchy” deterioration of a wetland, rather than the loss of a large area all at once. Different plant species have unique optimal growth ranges and tolerances (McKee and Mendelssohn 1989, Baldwin and Mendelssohn 1998), and may therefore succumb to biotic or abiotic stressors at different rates, resulting in the initial conversion of a fully vegetated wetland into one sparsely vegetated with signs of platform breakup and pond development (Hartig et al. 2002, Cahoon et al. 2019). The actual collapse event can be viewed as the point at which an area of wetland drops below the lower elevation limit for plant growth and persists into the subsequent years without natural recruitment of new emergent plants or an apparent response to natural morphodynamic feedbacks (Mudd et al. 2009, Fagherazzi et al. 2012). We further posit that true peat collapse is permanent (on an ecological timescale) and cannot revert back to a vegetated wetland platform without human intervention (e.g., the re‐establishment of elevation capital through restoration activities; Cahoon et al. 2019) or a significant shift in conditions (e.g., a major change in hydrology delivering new freshwater and sediment; Lewis et al. 2016). However, the limited time span of the existing studies (Table 1) make this unidirectional hypothesis difficult to validate, and at least one study (Cahoon et al. 2004) did document natural recolonization with a more flood‐tolerant species following the elevation drop, leading to the question of if this should be considered peat collapse.

With the establishment of a working definition, peat collapse can be differentiated from other forms of shallow subsidence in that, (1) the soils must have a high organic matter content, (2) there is a concomitant decrease in soil strength and structural integrity with the elevation loss, and (3) the resulting elevation and conditions are unconducive to natural recovery (i.e., an alternative stable state is established). For comparison, reports of elevation loss in the root‐zone of organic soils caused by drops in the water table and soil shrinkage (e.g., Whelan 2005, Cahoon et al. 2011) would not be peat collapse if the system naturally recovered within a few years. Shallow compaction due to the deposition of an overburden (e.g., Cahoon 2006) would not be considered peat collapse if soil strength (e.g., bulk density) actually increased during the elevation loss. Slow‐drowning, or the gradual decline in elevation capital in a coastal wetland that prevents it from keeping pace with sea level rise (e.g., Krauss et al. 2010, Lovelock et al. 2015b, Cahoon et al. 2019), would likewise not be peat collapse if soil strength and structure were never compromised, if the elevation loss was predominately deep (below the root zone), or the soils were not organic (roughly ≥20% organic matter by weight).

## Future Research Directions and Management Implications

Although this discussion strives to define and analyze not just why, but *how* peat collapse occurs in coastal wetlands, significant knowledge gaps remain to be filled before we can assess the contribution of peat collapse to coastal wetland loss. Specifically, mechanistic, controlled laboratory studies aimed at quantifying the sequential process of plant death, reduced soil strength and structural integrity, and elevation loss are needed to validate the conceptual framework and working definition presented herein. While our four proposed mechanisms of peat collapse agree with our current understanding of soil physics and biogeochemistry, direct empirical evidence is necessary to codify these concepts. Second, field manipulations are also needed, and should collect time‐series data that includes, at minimum, soil elevation, soil strength, bulk density, and mineralization rates as a function of depth and time since vegetation death. Site hydroperiod and salinity may also be key abiotic factors to monitor, as inundation duration and frequency will influence the probability of pore space and root channel collapse, vs. dilation, destabilization, and transport (Table 2). Third, areal change analysis (whether conducted via comparisons with historical aerial imagery or ongoing field‐based monitoring efforts) is needed to document vegetation health in conjunction with the magnitude and rates of vertical and lateral coastal wetland loss.

Unfortunately, many land managers and scientists are not aware a system is progressing toward peat collapse until wetland loss (submergence) has already occurred (Lewis et al. 2016). Key diagnostic indicators of vulnerability may include coastal wetlands with the following characteristics: (1) organic‐rich soils (≥ approximately 20% organic matter by mass, and an accompanying low bulk density and high porosity); (2) exposure to chronic stressors (salinity intrusion, altered hydroperiod, anthropogenic nutrient enrichment, etc.) or acute stressors (hurricanes, strong storms, lightning strikes, droughts, freezes, etc.); (3) indications of poor vegetation health (browning, thinning, or stunted growth).

Most importantly, land managers should focus efforts and resources on routine monitoring of coastal wetland vegetation so regions of declining plant health can be identified early, and the causes assessed and addressed before a regime shift toward open water occurs. Utilizing remote sensing and historical aerial imagery may be a cost‐effective approach, complemented by ground surveys, as needed. If newly emerging pockets of open water or regions of soil platform break‐up are found, a closer look at surficial soil structure may help identify the mechanism. An increase in soil bulk density within the rooting zone (relative to a healthy region, if no prior data are available) may be diagnostic of pore space compaction or root channel collapse in intertidal locations. At sites that are predominately subtidal, or remain saturated nearly continuously, a loose, flocculent layer above the marsh platform with reduced soil shear strength, may be observed. This suggests destabilization of waterlogged peat or mineralization, both of which result in mass loss of SOM. It is also important to note that shallow, open‐water pools in coastal wetlands can be a natural feature in the landscape (Wilson et al. 2010), making regular monitoring critical for differentiating a long‐term landscape feature from a regime shift related to vegetation death and possible peat collapse.

A major knowledge gap with significant implications for managers is how quickly the onset of peat collapse occurs, and whether the process is reversible. As mentioned, existing literature suggests elevation loss can occur anywhere from 5 months to 3 yr following a disturbance event that severely damages vegetation health. We view this as the critical period for action in which managers should seek to remove all stressors from the system and potentially intervene with restoration and rehabilitation efforts to restore vegetative cover. Once elevation loss has been initiated and interior ponds form, hydrologic forces can expand the size of the pond through “bank slumping” (Stevenson et al. 1985) or “soil creep” (Mariotti 2016). These mass wasting processes begin when interior ponds reach a critical width (which varies according to the local tidal range), causing edge soils to move downslope by gravity, slowly expanding the pond (Mariotti 2016, Mariotti et al. 2016). Restoration techniques such as thin layer placement of dredge material have been successful at coastal wetland locations with an elevation deficit (Croft et al. 2006, VanZomeren et al. 2018), but to our knowledge have not been applied specifically to sites of peat collapse.

## Conclusions

Peat collapse is a term invoked sporadically to describe elevation loss in a variety of organic‐rich soils, from polar/boreal wetlands to raised bogs, and is becoming increasingly used in coastal wetland discussions and literature. Through an analysis of existing peer‐reviewed literature and a thorough understanding of organic wetland soil biogeochemistry and physics, we propose that coastal wetland peat collapse is a type of shallow subsidence (i.e., elevation loss occurring within the active root zone) unique to organic soils that is initiated by severe stress or death of the existing plant community. Following root decline, aerenchyma contracts and the loss of the structural support provided by live roots allows for possible pore space or root channel compression, SOM swelling and deconsolidation, or accelerated mineralization. Without significant autotrophic C sequestration, the area becomes a net C source, which may be further exacerbated if the site is also experiencing saltwater intrusion, nutrient enrichment, or decreased water level. The elevation loss is likely to manifest within 5 months to 3 yr of vegetation decline and could range from 1 to 15 cm. Additional research is needed to experimentally test the validity of each proposed mechanism of coastal wetland peat collapse. Management efforts should focus on landscape‐scale monitoring of vegetation health as a precursor to elevation loss. Moreover, as new research investigates processes and case studies related to coastal wetland peat collapse, it is critical that terminology be codified and consistently applied so the knowledge base can be expanded and made easily accessible to new researchers.
